# Aprotic Electrolytes Beyond Organic Carbonates: Transport Properties of LiTFSI Solutions in S‐Based Solvents

**DOI:** 10.1002/cssc.202402273

**Published:** 2025-01-17

**Authors:** Vanessa Piacentini, Cataldo Simari, Emanuela Mangiacapre, Adriano Pierini, Antonio Gentile, Stefano Marchionna, Isabella Nicotera, Sergio Brutti, Enrico Bodo

**Affiliations:** ^1^ Department of Chemistry Sapienza University of Rome P.le Aldo Moro 5 00185 Rome Italy; ^2^ Department of Chemistry University of Calabria 87036 Arcavacata di Rende (CS) Italy; ^3^ Ricerca sul Sistema Energetico – RSE S.p.A. Via R. Rubattino 54 20134 Milano Italy; ^4^ CNR-ISC Consiglio Nazionale Delle Ricerche Istituto Dei Sistemi Complessi 00185 Rome Italy; ^5^ GISEL - Centro di Riferimento Nazionale per i Sistemi di Accumulo Elettrochimico di Energia 50121 Florence Italy

**Keywords:** aprotic electrolytes, Lithium batteries, sulphones, sulphoxides, LiTFSI

## Abstract

This work illustrates a physico‐chemical study of the structural, dynamic, and transport properties of electrolytes made of LiTFSI solutions in sulphoxide and sulphone solvent mixtures. Experimental measurements, by Raman and NMR spectroscopies, as well as electrochemical impedance spectroscopy, reveal the formation of a variety of ionic aggregates depending on the solvent composition that significantly affect the ion mobility and conductivity of the electrolyte. Mixtures containing tetrahydrothiophene‐1‐oxide exhibit a larger ion mobility due to a rapid exchange mechanism between solvent molecules, whereas the use of tetramethylene sulphone favors the formation of ionic aggregates due to the strong dipolar interactions between solvent molecules.

## Graphical abstract

Extensive physico‐chemical characterizations of electrolytes obtained by dissolution of LiTFSI in sulphone‐based solvents and their mixtures are presented. Their conductive properties are discussed in connection with the structural description provided by Raman spectral evidence.

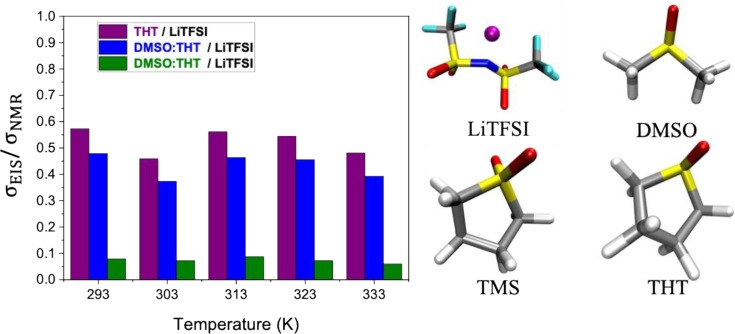

## 1 Introduction

The design and optimization of batteries are crucial steps for the future. This is an extremely complex challenge, as these systems consist of many interconnected components, each of which can be formulated in different ways. Among these components, the electrolyte plays a critical role in determining electrochemical performance and the operating and storage temperature ranges. Aprotic electrolytes have emerged as dominating components of any commercial advanced battery systems. Given the specific interfaces forming at both electrodes sides upon cycling, these electrolytes must meet numerous stringent criteria,^[1]^ encompassing bulk composition, ion transport, and interfacial stability, while also considering aspects such as viscosity, safety, toxicity, and cost. The selection of the solvent component of the electrolyte is particularly significant, as it impacts charge transport and adhesion to active materials, thereby influencing wetting capability.[^2^, ^3^]

The use of sulphones and sulphoxides in aprotic electrolytes for batteries represents a promising approach to enhancing battery performance. These compounds may contribute to improve transport properties, to enhance stability and safety as well as to alter the usual de‐solvation thermodynamics across the electrode/electrolyte interface in battery systems.

Dimethyl sulphoxide (DMSO) is an aprotic dipolar solvent that incorporates a strongly polar group and two hydrophobic methyl groups. Due to its properties, such as low toxicity and environmental compatibility, DMSO has been and still is widely used as a solvent in industry and scientific research, finding applications in fields as distinct as biochemical, pharmacological technology and electrochemistry. DMSO is generally used in conjunction with other substances with which it interacts on a molecular scale through hydrogen bonds (with water, for example) and electrostatic forces (with dipole‐permanent molecules). These interactions determine the dissolution properties of charge transport species and the overall macroscopic behavior of the resulting electrolyte and exert an influence on the molecular structures of the molecules/ions involved. Going beyond DMSO, many organic sulphones and sulphoxides have attracted significant attention for their potential to improve battery characteristics.^[4]^

The incorporation of sulphones and sulphoxides in aprotic electrolytes for batteries provides several advantages. These compounds directly impact the liquid phase physical properties, solvate structures, and ion transport properties, all of which are critical factors for efficient battery operation.^[4]^ Additionally, these compounds can affect the deprotonation processes in electrolytes, essential for catalyzing parasitic reactions within the battery system.^[5]^ Furthermore, sulphones and sulphoxides play a role in mediating surface processes, thus contributing to an enhanced galvanostatic efficiency in batteries.^[6]^ As an example, the redox behavior of Cu_2_S as active electrode in aprotic electrolytes can be tuned by the presence of sulphones and sulphoxides in the electrolyte formulation, suggesting the use of these compounds to enhance the performance of rechargeable Li−S batteries.^[7]^

The use of mixed electrolytes containing sulfolane and other components is also beneficial in the enhancement of electrolytes stability and ionic transport in aprotic Li‐O_2_ batteries^[8]^ where the formation of Li_2_O_2_ and its role in determining the kinetics of oxygen reduction and evolution reactions are crucial for battery performance.^[9]^ Specifically, the study of the influence of the aprotic electrolyte composition on the mass transfer processes in positive electrodes in Li‐O_2_ batteries is vital for optimizing battery efficiency and longevity.^[10]^ Additionally, the knowledge of the structural characteristics, diffusion properties, and stability of lithium salts in aprotic electrolytes allows to disentangle the complex interplay among different components in battery systems.^[11]^

Based on these considerations, our work aims for the first time to provide an extensive physico‐chemical characterization of lithium bis(trifluoromethanesulfonyl)imide (LiTFSI) in solutions containing DMSO mixed with molecules characterized by similar chemical and physical properties, such as tetramethylene sulphone (TMS) and tetrahydrothiophene‐1‐oxide (THT). These species have been previously used in lithium‐ion batteries both as additives to increase the oxidation potential of the electrolyte and or as co‐solvents and has been recently characterized by us using molecular dynamics simulations,^[12]^ but a clear comprehension of the interplay between liquid phase structure and functional properties is missing.[^13^, ^14^, ^15^]

In particular, our study will focus on three electrolyte formulations: THT/LiTFSI 1 m, DMSO:THT (50:50 v/v)/LiTFSI 1 m and DMSO:TMS (50:50 v/v)/LiTFSI 1 m, which can potentially emerge as economical and non‐toxic alternatives to traditional carbonate‐ and ether‐based electrolytes. Both THT and TMS have a high boiling and flash point. TMS is a solid at room temperature (r.t.) and, although it melts at 27.5 °C, we have chosen to use it as a co‐solvent in a mixture with DMSO to ensure its applicability over a wider temperature range.[^16^, ^17^] In contrast, THT, which is liquid at room conditions, has been evaluated both as a co‐solvent with DMSO and in its pure state.

## 2 Methodology

### 2.1 Electrolytes Preparation

DMSO [dimethyl sulfoxide anhydrous,≥99 %], THT [Tetrahydrothiophene 1‐oxide, 96 %], TMS [tetramethylene sulfone, 99 %] were purchased from Sigma Aldrich. All solvents were purified over 3 Å molecular sieves for a week prior to use, except for TMS, which is in a solid state at room temperature and was used as received. LiTFSI (Lithium bis(trifluoromethanesulfonyl)imide extra dry <20 ppm H_2_O, was purchased from Solvionic and dried at 120 °C under vacuum for 12 hours. The formulations with the solvents blend were prepared as 50 : 50 v/v. The electrolytes with 1 mol kg^−1^ LiTFSI, were prepared in an argon‐filled glovebox (H_2_O and O_2_ contents<1 ppm, MBRAUN). The corresponding molarities of the three electrolytes at 293 K are 0.97, 0.95 and 0.99 for THT, THT/DMSO and TMS/DMSO respectively.

### 2.2 Density Measurements

The determination of density (ρ) values was conducted using a Mettler Toledo Density Meter DM45 DeltaRange within the temperature range of 293 to 333 K. Prior to measurement, a calibration process was performed using dry air and bi‐distilled water at 293 K.

### 2.3 Viscosity Measurements

The dynamic viscosity (η) values were obtained using an Anton Paar micro viscometer (model Lovis 2000 M/ME, with an accuracy of up to 0.5 %), employing Peltier elements (accuracy of 0.02 K) and calibrated glass capillaries of varying diameters (1.59 mm, 1.80 mm, and 2.50 mm, depending on the targeted viscosity range). The measurements were conducted within a temperature range from 293 to 333 K.

### 2.4 Raman Spectroscopy

Raman spectra were carried out in a Horiba XploRA PLUS system equipped with an Olympus BX43 microscope and a long working distance 50x (NA=0.50) objective. We used a 532 nm solid state laser and 2400 line/mm diffraction grating to collect the spectra, that combined with the resolution (1024x256 pixels, pixel dimension 26x26 μm^2^) of air‐cooled Peltier CCD detector assure ≈1 cm^−1^ spectral sensitivity. To preserve the degradation of the samples during the measurements, all the sample are stored in specific holder sealed in inert atmosphere and the power of laser was kept in the 5–8 mW range. To have a more complete view of the Raman shift of our organic samples, all the spectra was collected in the range from 60 to 3200 cm^−1^ at room temperature.

### 2.5 NMR Spectroscopy

NMR measurements were conducted using a Bruker AVANCE 300 NMR Wide Bore spectrometer, operating at 300 MHz on ^1^H, 116.6 MHz on ^7^Li, and 282.4 MHz on ^19^F nuclei, respectively. A Diff30 Z‐diffusion 30 G/cm/A multinuclear probe with substitutable RF inserts was employed. Self‐diffusion coefficients and spin‐lattice relaxation times were acquired on ^1^H for solvent molecules, ^7^Li for Li^+^ cations and ^19^F for TFSI^−^ anions, respectively, across a range of temperatures spanning from 20 °C up to 60 °C, with increments of 10 °C. At each temperature, the samples were allowed to equilibrate for approximately 20 minutes. To prevent any contact with moisture, the NMR samples were prepared in glovebox and hermetically sealed in 5‐mm Pyrex tubes.

For the self‐diffusion coefficient measurements, the pulsed field gradient stimulated echo (PFG‐STE) method was used.^[18]^ The sequence involves three 90° RF pulses (π/2‐τ1‐π/2‐τm‐π/2) and two gradient pulses that are applied after the first and the third RF pulses, respectively. The resulting echo is registered at time τ=2τ1+τm. Following the standard notation, the magnetic field pulses are characterized by their magnitude (g), duration (δ), and time delay (▵). The Fourier‐transformed echo decays were analyzed using the Stejskal–Tanner expression:
(1)

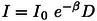




Where the parameters I and I_0_ represent the intensity or area of selected resonance peaks in the presence and absence of gradients, respectively. Meanwhile, β signifies the field gradient parameter, defined as β=[(γgδ)^2^×(▵−δ/3)] and D is the measured self‐diffusion coefficient. The experimental parameters employed for the investigated samples were: δ=0.8–2 ms, time delay ▵=8–20 ms, and the gradient amplitude varied from 100 to 950 Gauss cm^−1^. With the low standard deviation observed in the fitting curve and the repeatability of the measurements, the uncertainties in the self‐diffusion measurements are approximately 3 %. Lastly, longitudinal or spin‐lattice relaxation times (T_1_) were determined using the inversion‐recovery sequence (π–τ − π/2).

### 2.6 Electrochemical Impedance Spectroscopy (EIS)

The electrochemical impedance spectra were recorded to determine the ionic conductivity of electrolytes, using Biologic electrochemical workstation (model VSP) in the temperature range of 293–333 K and in the frequency range between 10 kHz and 10 Hz with an applied potential of 10 mV. This analysis enables the determination of the electrolyte‘s resistance value. At elevated frequencies, extracting the bulk resistance value becomes feasible through curve interpolation, utilizing the value derived from the real component of impedance along the abscissa axis. The conductivity is derived via the following below:[^19^, ^20^]

(2) $$\sigma =\frac{1}{R}\cdot \frac{L}{S}\;$$

where R is the ohmic resistance, L and S represent the distance between the blocking electrodes and the electrode area, respectively.

The analysis was performed with a coin cell in a symmetric configuration with two stainless steel blocking electrodes (16 mm diameter). A Teflon disc measuring 16 mm in diameter and 2.15 mm in thickness was placed between the two electrodes. The disc was then punched at the center to create a hole with a diameter of 6 mm, into which the electrolyte was inserted.

### 2.7 Density Functional Theory Calculations

To clarify the vibrational components of the Raman bands associated with the S−N−S vibration of the TFSI anion, a set of calculations on 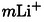 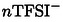 clusters were performed by us because the interpretation of the spectra from literature data turned out to be uncertain. For each cluster with different *m* and *n*, a certain number of initial configurations were generated and subsequently optimized using the low‐cost semiempirical GFN2‐xTB^[21]^ method. The lowest‐energy structure for each cluster was re‐optimized using the ωB97XD functional^[22]^ in combination with the Sadlej‐pVTZ basis set,^[23]^ especially tailored to accurately reproducing the Raman intensities. Theoretical Raman spectra were computed for the isolated anion, 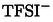 (in both cis‐C_1_ and trans‐C_2_ conformations) and for the following clusters: ${\mathrm{Li}}^{+}{\mathrm{TFSI}}^{-}$ , $2{\mathrm{Li}}^{+}{\mathrm{TFSI}}^{-}$ , $3{\mathrm{Li}}^{+}{\mathrm{TFSI}}^{-}$ , ${\mathrm{Li}}^{+}2{\mathrm{TFSI}}^{-},$ $2{\mathrm{Li}}^{+}2{\mathrm{TFSI}}^{-}$ , and $3{\mathrm{Li}}^{+}2{\mathrm{TFSI}}^{-}$ . The computed gas‐phase Raman spectra in the S‐N‐S vibration region are shown in Figure S5 d. The theoretical vibrational band positions were scaled by a factor of 0.97 to correct for anharmonicity.^[24]^ All calculations were performed using Gaussian16. Bulk solvent effects were not included in this set of calculations.

### 2.8 Electrochemical Stability Window

Linear Sweep Voltammetry (LSV) and Cyclic Voltammetry (CV) were used to evaluate the electrochemical stability window (ESW) of electrolytes. The ESW was studied using LSV and CV measurements at a scan rate of 1 mV s^−1^ in high‐potential and low‐potential ranges, respectively. The LSV measurement was conducted from the open circuit voltage (OCV, approximately 3 V) up to around 6 V versus Li^+^/Li, while the CV test was performed in the potential range between 0.001 V and 2.5 V versus Li^+^/Li. The LSV and CV measurements were carried out using a BioLogic potentiostat/galvanostat. Measurements were carried out in an ECC Std EL‐CELL test cell with two electrode configurations. A lithium metal foil (thickness 0.6 mm, purity 99,9 %, acquired from Merck) was utilized as counter‐electrode, which was punched into a disc shape with a diameter of 10 mm. A glass fiber separator Whatman® (1.55 mm thickness, 18 mm diameter), soaked in 150 μ l of electrolytes was used. To prevent parasitic reactions, a Platinum inert planar electrode was used as the working electrode (diameter=10 mm). The choice of platinum as the working electrode avoids the activation of parallel chemical reactions that could involve other electrode components, such as SuperP carbon or ligands.^[25]^ In this way, electrolyte degradation processes can be revealed by analyzing the amount of charge transferred.

## 3 Results and Discussion

### 3.1 Physical Characterization

**Figure 1:**
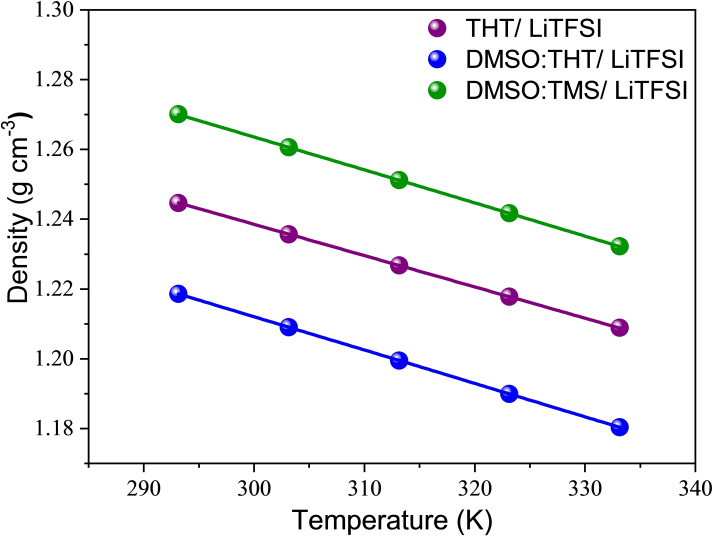
Density values of the probed electrolytes. Solid lines represent the fitting in terms of the model described in the eq. (3).

Bulk densities for the liquid electrolytes are shown in the Figure 1 in the temperature range from 293 to 333 K. For all electrolytes, a second‐order polynomial equation^[26]^ was used to fit the behavior of density as a function of temperature (eq. (3)), where A, B and ρ_0_ represent the fitting parameters (Table S1.

(3) $$\rho \left(T\right)=\rho_{0}+BT+AT^{2}$$

The thermal expansion observed in the samples as temperature increases can be accurately quantified using the isobaric thermal expansion coefficient,^[26]^ α_p_, which is defined as:

(4) $$\alpha_{P}=-\frac{1}{\rho \left(T\right)}\cdot {\left(\frac{\delta \rho \left(T\right)}{\delta T}\right)}_{P}$$

Considering the quadratic model used for the ρ(T), this coefficient can be determined as :

(5) $$\alpha_{P}=\frac{-(2AT+B)}{AT^{2}+BT+\rho_{0}}\;$$

The calculated thermal expansion coefficients for all the electrolytes are presented in Figure S1. We observe a monotone increase with temperature in all the investigated cases, with the slope becoming steeper as the DMSO is added. Comparing slopes (Table S2), it can be seen that the DMSO:TMS mixture is more affected by the temperature variation, followed by DMSO:THT and finally THT. Experimental dynamic viscosity values η (mPa⋅s) of probed mixtures were obtained in a temperature range from 293 to 333 K.

**Figure 2:**
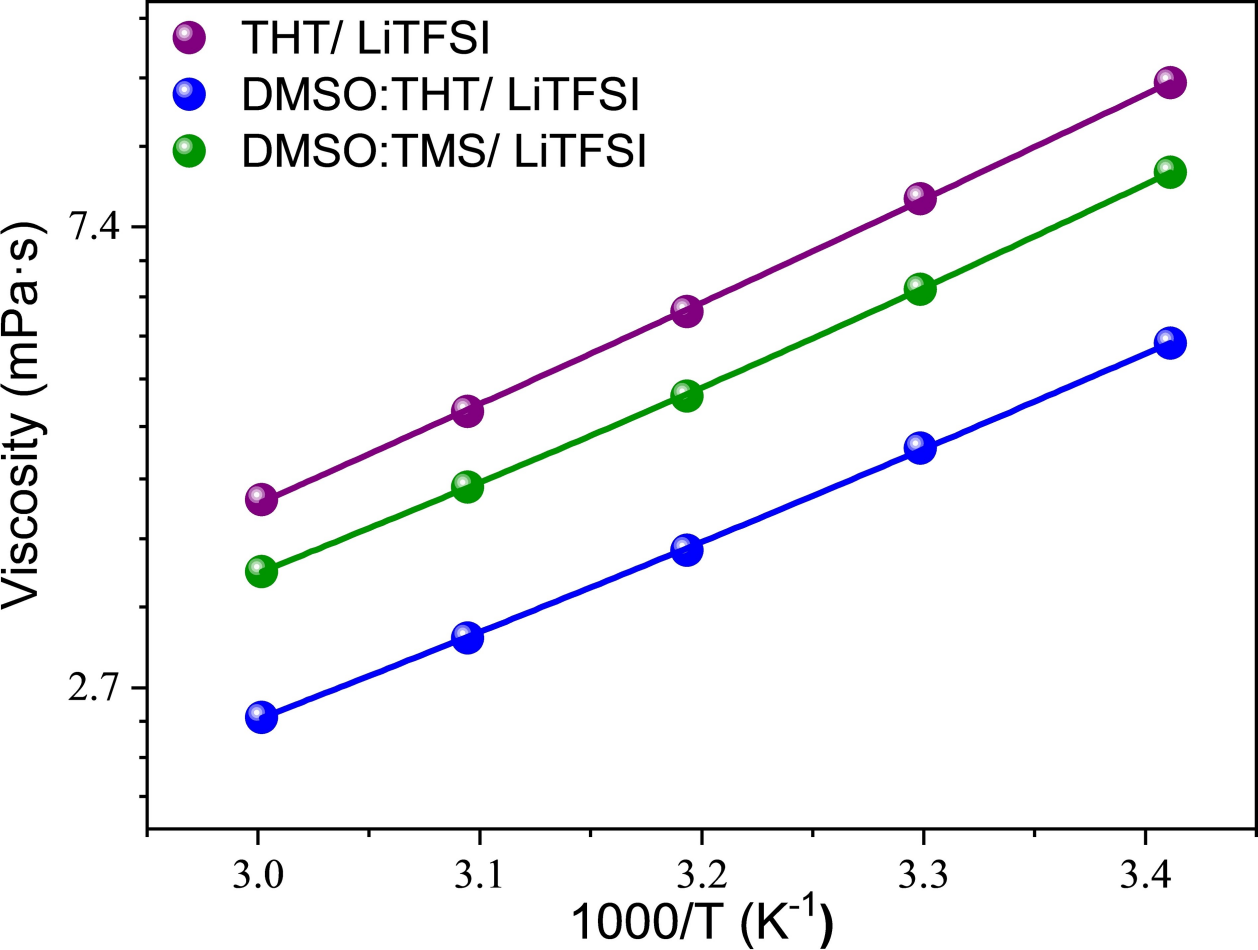
Dynamic viscosity η (mPa ⋅ s) of the probed mixtures as a function of temperature. Solid lines represent the fitting in terms of the VFT model.

As expected, viscosity decreases at higher temperature as shown in Figure 2. The temperature‐dependence of the data can be fitted using the Vogel‐Fulcher‐Tammann[^27^, ^28^, ^29^, ^30^, ^31^, ^32^, ^33^] (VFT) method, as   in eq. 6.

(6) $$ln\left(\eta \right)=ln\left(\eta_{0}\right)+\frac{B}{T-T_{0}}\;$$

where η_0_ is the value of the viscosity at the high‐temperature limit, B is a quantity related to the activation barrier, and T_0_ is the so‐called “Vogel temperature” at which the configurational entropy is zero.

The obtained fitting parameters are summarized in the table S3 and have been used to estimate the activation energy for viscous flow, E_a,η_, from the follow relationship:

(7) $$E_{a,\eta }=R\frac{d\;ln\left(\eta \right)}{d\;\left(\frac{1}{T}\right)}=RB{\left(\frac{T}{T-T_{0}}\right)}^{2}\;$$

where R is the gas constant. The energy barrier that a molecule or ion must overcome in the viscous flow is $E_{a,\eta }$ and is related to the minimum energy required for molecules to move past each other: the higher the activation energy, the more difficult it is for ions to move in the fluid.^[34]^ Figure S2 shows the values of $E_{a,\eta }$ for each system as a function of temperature. It is evident that the barrier is lower for the DMSO:THT solution at all temperatures, indicating that ions can move more freely compared to the other solutions. For the THT formulation, this value is less dependent on temperature, as the trend remains constant, indicating strong ionic interactions. In contrast, for the DMSO:TMS solution, temperature significantly influences local motions, leading to a variation in the liquid structure. Specifically, $E_{a,\eta }$ varies by about 3.25 kJ mol^−1^ from 293 to 333 K.

For the sake of completeness, the ESW of the electrolytes has been measured as shown in the Figure S3 that shows the CV and LSV profiles. In the case of THT/LiTFSI electrolyte, the expected irreversible cathodic and anodic features are observed during the first cycle whereas these signals fade in the following cycles. This evidence suggests that a passivating film is forming on the electrode surface, mainly in the first cycle. The mixed DMSO:THT/LiTFSI electrolyte shows features in line with the THT/LiTFSI electrolyte with a slightly higher onset potential for the anodic degradations. In contrast, the DMSO:TMS/LiTFSI electrolyte shows a weak irreversible cathodic feature at 1.5–1.6 V vs Li, much smaller compared to both THT/LiTFSI and DMSO:THT/LiTFSI, thus possibly indicating a better ability to form a stable passivation film. Overall, the use of TMS appears to have a stabilizing effect, limiting solvent decomposition and the formation of unwanted by‐products.

### 3.2 Raman Analysis

Raman spectroscopy has been employed to investigate the vibrational features of DMSO, THT, TMS, and LiTFSI in their pure states, in their mixture with co‐solvents and in the electrolyte solutions, in the range from 60 to 3200 cm^−1^. The solvent and solute structures are affected by the physical properties of the molecules, such as their shape, polarity, and configurations. Previous studies[^35^, ^36^, ^37^] have shown that dipolar aprotic molecules with extremely large dipole moments exhibit strongly non‐ideal physicochemical behaviors due to their tendency to form intermolecular aggregates with themselves and with other molecules with similar dielectric properties. Various experimental techniques,[^35^, ^37^, ^38^] along with theoretical and computational studies,^[36]^ suggested that neighboring sulfoxide molecules predominantly align in an antiparallel configuration due to dipole‐dipole electrostatics between the S=O groups. These findings strongly suggest that these molecules have a noticeable tendency to form dimers, both in the pure liquid state and in solution.

**Figure 3:**
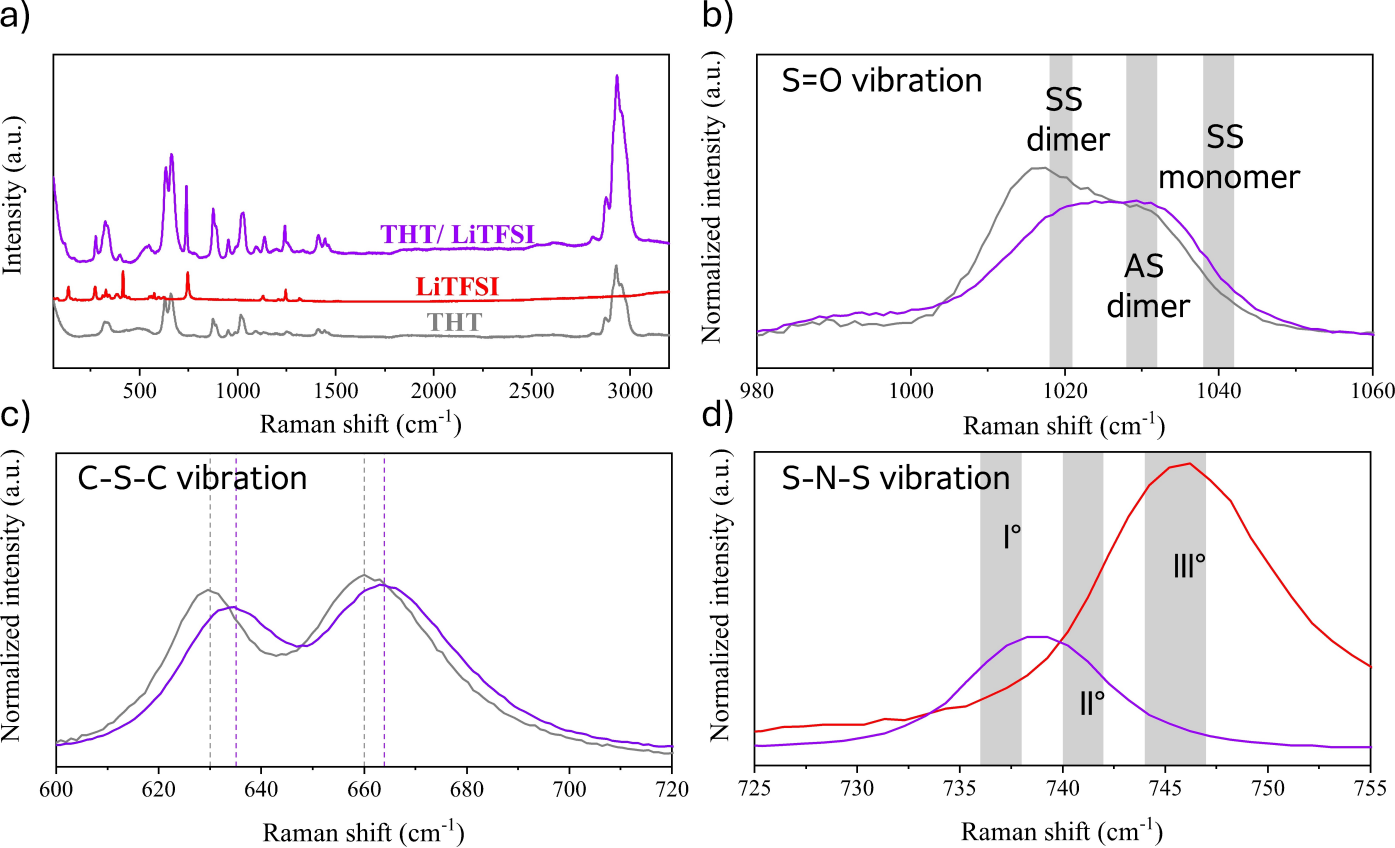
a) Raman spectra of pure THT solvent (grey line), LiTFSI salt (red line), and the 1 m electrolyte solution (purple line); b) the S=O stretching region of THT in pure (grey) and electrolyte solutions (purple); c) C−S−C stretching region (SS and AS, respectively at low and high frequencies) of THT in pure (grey) and electrolyte solution (purple); d) S−N−S vibration region of pure TFSI^−^ (red) and in the electrolyte solution (purple), with the intervals corresponding to the positions of the main band‘s components marked in grey (see text).

**Figure 4:**
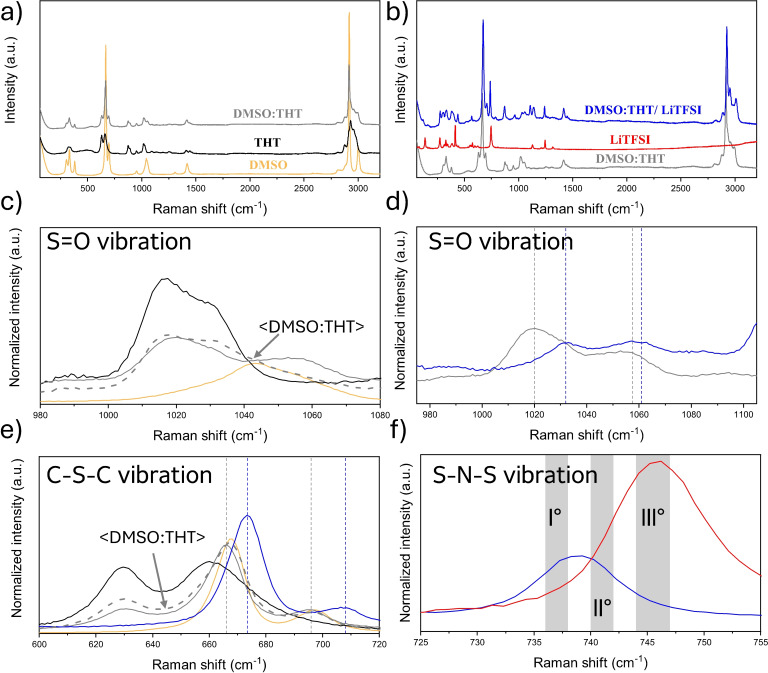
a) Raman spectra of pure DMSO solvent (yellow line), THT solvent (black line) and DMSO:THT mixture (grey line); b) Raman spectra of DMSO and THT mixture (grey line), LiTFSI salt (red line), and the 1 m electrolyte solution (blue line); c) the S=O stretching region of THT and DMSO in neat and mixture solutions (the arithmetic average of DMSO and THT spectra is reported with grey dashed line); d) S=O stretching region in the DMSO:THT mixture (grey) and electrolyte solutions (blue); e) C−S−C stretching region of neat THT (black), neat DMSO (yellow), DMSO:THT mixture (grey) and electrolyte solution (blue), the arithmetic average of DMSO and THT spectra is reported with grey dashed line; f) S−N−S vibration region of pure TFSI^−^ and in the electrolyte solution, with the intervals corresponding to the positions of the main band‘s components marked in grey (see text).

**Figure 5:**
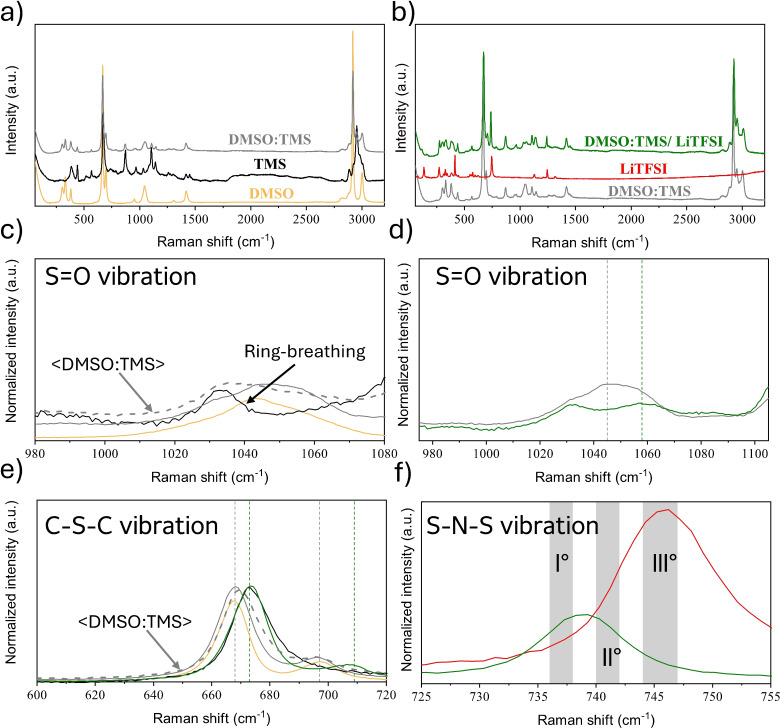
a) Raman spectra of pure DMSO solvent (yellow line), TMS solvent (black line) and DMSO and TMS mixture (grey line); b) Raman spectra of DMSO:TMS mixture (grey line), TFSI (red line), and the 1 m electrolyte solution (green line); c) S=O stretching region of TMS (black), DMSO (yellow) and mixture (grey); the arithmetic average of DMSO and TMS spectra is reported with grey dashed line; d) S=O stretching region of TMS in mixture (grey) and electrolyte solutions (green); e) C−S−C stretching region of neat TMS (black), neat DMSO (yellow), DMSO:TMS mixture (grey) and electrolyte solution (red), the arithmetic average of DMSO and THT spectra is reported with grey dashed line; f) S−N−S vibration region of pure TFSI^−^ (red) and in the electrolyte solution (green), with the intervals corresponding to the positions of the main band‘s components marked in grey (see text).

In Figures 3, 4 and 5, Raman spectra and their respective magnifications of the three electrolytes and of their components are reported. Figure 3a, shows the spectra of the neat THT solvent, of the solid LiTFSI salt, and of the 1 m electrolyte solution. Similarly, Figures 4a, 4b, 5a and 5b show the spectra of neat DMSO, of the neat co‐solvents (THT and TMS), of their mixture (DMSO:THT and DMSO:TMS) and the spectra of the LiTFSi 1 m electrolytes.

The interpretation of the Raman spectral region corresponding to the S=O group vibration (Figure 3b) is relatively simple, involving the S=O stretching frequency of monomers and dimers, with the latter split into a symmetric (SS) and an asymmetric stretching (AS) contribution (they are indicated by the three grey regions in Figure 3b). In the electrolyte solution (purple line), the stretching mode signal is blue‐shifted, and we can also note a reduced intensity of the dimer component, indicating that the salt presence tends to break the alignment of the S=O dipoles with respect to the pure liquid bulk state (grey line). As a consequence, the strength of the S=O bond increases in the electrolyte compared to the neat solvent case, indicating a restructuring of the solvent configurations promoting lithium‐ion coordination. This implies that the strong dipolar interactions fade in favor of the electrostatic intermolecular interaction with the salt.

Turning to the other S‐based solvents, the picture becomes more complex due to the increasing number and variety of intermolecular interactions, as these molecules can also interact through dipole‐dipole forces. Specifically, the mixing of THT and DMSO (Figure 4c and d) results in an only slightly perturbed THT (the signal around 1020 cm^−1^ is similar), but a blue‐shifted S=O stretching band for the DMSO (from 1040 to 1060 cm^−1^). The electrostatic network of dipole‐dipole interactions of DMSO is evidently disrupted by the presence of TMS, although the two molecules tend to self‐aggregate as shown by the relatively small difference between the arithmetic average of the pure solvent spectra (grey dashed line) and the experimental spectra of the mixture (solid grey line). The difference between the two curves between 1040 and 1080 cm^−1^ highlights the presence of a limited degree of dipolar interactions between the different solvent molecules. Figure 4d shows the spectral region of the S=O peaks of THT and DMSO in the presence (blue line) and absence (grey line) of the salt, confirming the impact of solvation that broadens the peaks corresponding to the stretching modes due to the strong dipole‐charge interactions between both S=O groups toward the salt ions.

The Raman spectra for TMS is shown if the Figure 5 in an analogous fashion to THT (see the Figure 4). The analysis is more complex due to the appearance in the S=O stretching region of a feature originated by a low energy conformer of TMS that corresponds to the ring breathing mode.^[39]^ In the S=O stretching region of the DMSO, TMS and their mixture (Figure 5c and d), only the DMSO band is observed and, once again, a shift towards higher wavenumbers can be seen in both in the DMSO:THT mixture (Figure 5c) and in the presence of the salt (Figure 5d). These effects are in line to what already observed for THT (see above). For TMS, a slight blue shift of its S=O signals can be observed for the bands related to the symmetric stretching of SO_2_ between 1105 and 1140 cm^−1^ (Figure S4 a).[^16^, ^39^] Turning to the full electrolytes formulation a clear interpretation of the spectra is highly challenging since the band convolution of the SO_2_ stretching bands of TFSI and TMS results in substantial broadening of the signals (Figure S4 b).

In Figures 3e, 4e and 5e, we also report a magnification of the 600–720 cm^−1^ region of the spectra, where the vibrations of the C−S−C stretching modes of the solvent molecules can be observed. For mixed‐solvent formulations, the Raman spectra of the solvent mixtures closely match the arithmetic average of the pure solvents, suggesting that this band is largely unaffected by mixing. On the other hand, the vibrational modes in this region for cyclic molecules correspond to ring‐breathing modes.^[39]^ Turning to the electrolyte formulations, in all systems the addition of LiTFSI results in a blue shift of the bands that can be attributed to the coordination of solvent molecules with LiTFSI.

As a final point of the vibrational analysis of these formulation, we focus on a specific vibrational feature of the LiTFSI salt..[^8^, ^40^, ^41^] Generally speaking, due to the charge delocalization in the TFSI^−^ anion, the interaction between lithium and the anion is not strong, making it soluble in a wide range of solvents with varying physicochemical properties. However, the assumption of weak coordination between the TFSI^−^ anion and metal cations has been challenged by studies that demonstrate significant contact ion pairing with Li^+^ in various solvents, such as acetonitrile and glymes, even at relatively low concentrations.[^40^, ^42^, ^43^] Several studies[^40^, ^44^, ^45^, ^46^] have reported that the most representative band related to the behavior of TFSI^−^ is associated with the S−N−S vibrational mode, which in its crystalline state is found around 747 cm^−1^. When Li^+^ cations coordinate with solvents, this vibrational band shifts due to changes in the anion′s electron density and structure. For formulations with concentrations comparable to those used in this study, this band shifts to around 740 cm^−1^.

To study the coordination behavior of the TFSI anion, the S−N−S vibrational band becomes a valuable tool to examine the degree of local confinement and coordination of the TFSI anion in electrolytes. Several studies[^42^, ^46^, ^47^] have identified three region within this band, attributing each component to different coordination states of the TFSI anion: 736–738 cm^−1^ correspond to the presence of “free” solvated TFSI, 740–742 cm^−1^ corresponds to solvent‐separated ion pair (SSIP) structures, 744–747 cm^−1^ corresponds to contact ion pair (CIP) or ionic aggregates (AGG).^[46]^ This assignment is based on a study[^40^, ^42^] in which Raman spectra of crystalline solvates of LiTFSI were correlated with known crystal structures. It is important to note that, in the liquid phase, the bands are slightly shifted due to the structural flexibility of the TFSI anion, which can assume different conformations. Each conformation can coordinate differently to one or more Li^+^ cations, thus affecting the position of the Raman bands.

To clarify the assignment of the bands, a theoretical study on 
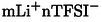
 clusters was conducted (see Section S2), with varying composition and charge states (*m*=0–3 and *n*=1–2), using gas‐phase DFT calculations. From the computed data, it can be deduced that the main components of the experimental Raman spectra (component I and II in Figures 3d, 4 f and 5 f, red and blue lines in Figure S5 a–c) appear to be due to the cationic structures 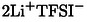 (735 cm^−1^) and 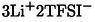 (740 cm^−1^). The third component (green line in Figure S5 a–c) is essentially negligible and likely due neutral forms such as 
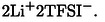
 The latter aggregate also contributes to the low wavenumber portion of the absorption band together with other neutral aggregates and free 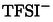 (720 cm^−1^). A notable trend is that aggregates with a single positive charge, i. e., systems with one more Li^+^ than TFSI^−^, tend to contribute to the components at higher Raman shifts (see the discussion of Figure S5 in the SI).

From the deconvolution of the experimental spectrum of the S−N−S band for the three systems (Figure S5 a–c), it is evident that the electrolyte with TMS as a co‐solvent exhibits a higher fraction of components 2 and 3. The order of electrolytes, ranked from those with the highest number of aggregates to those with the lowest, is as follows: DMSO:TMS>THT>DMSO:THT. This result is very interesting because previous studies have shown that TMS is used as an additive or co‐solvent to reduce interactions between the cations and anions of the salt. It can therefore be inferred that the strong dipolar interactions between the different solvent molecules can hinder this effect.[^8^, ^48^, ^49^]

### 3.3 Multinuclear NMR Characterization and EIS

**Figure 6:**
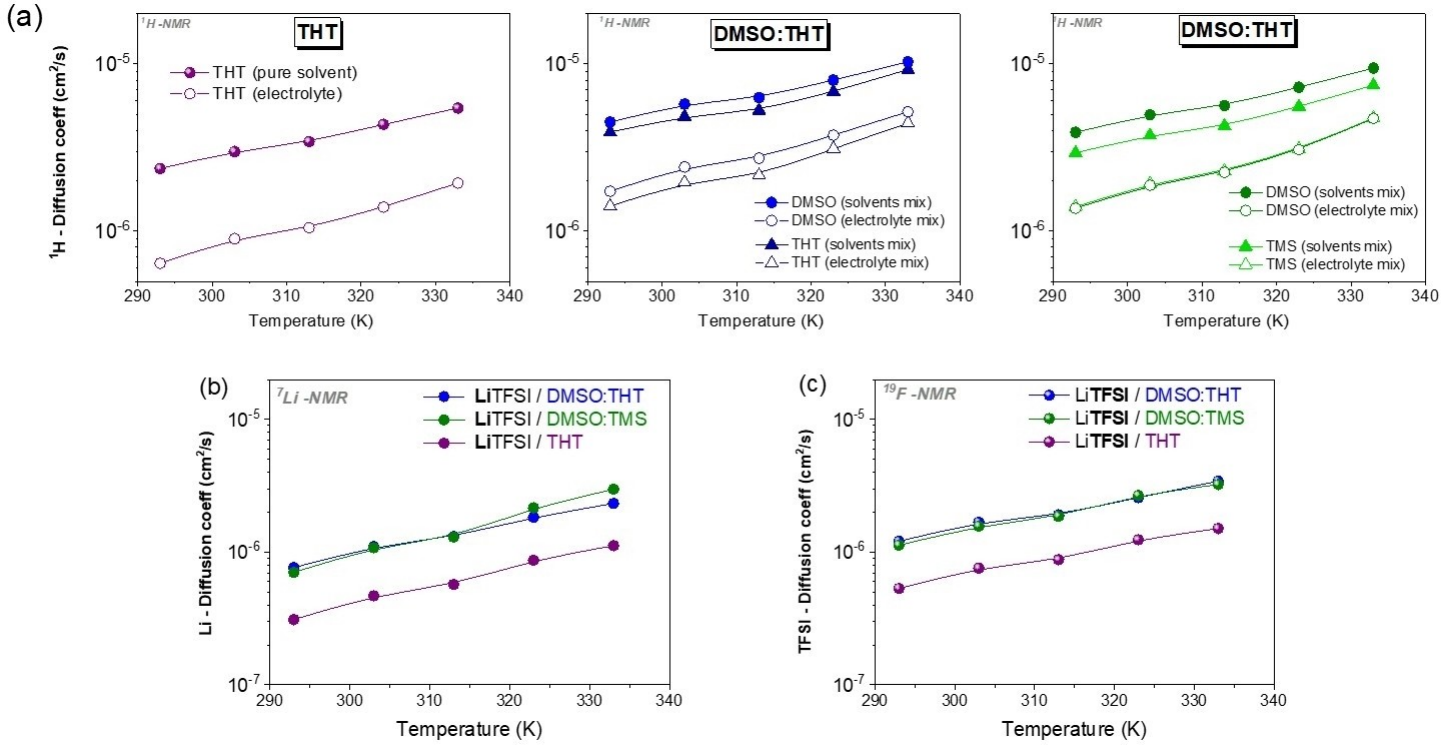
Temperature dependence of the self‐diffusion coefficients of: a) solvent molecules without (solvents mix) or with (electrolyte mix) addition of LiTFSI salt; b) Lithium cations in electrolytes and c) TFSI anions in electrolytes.

The self‐diffusion coefficients of all the species present in the three electrolyte solutions, solvents (^1^H), Li^+^ ion (^7^Li), and TFSI^−^ (^19^F), were measured by PFG‐STE NMR spectroscopy, and their temperature dependence is shown Figure 6. The findings of this NMR investigation are as follows:

1. the diffusivity of the solvent molecules decreases following the dissolution of the LiTFSI salt. Clearly, this result is ascribed to the direct involvement of the organic molecules in the solvation of salt ions. It is worth noting that the greatest reduction is observed in the case of pure THT, suggesting a strong structuring of the electrolyte solution.
2. the diffusion coefficients for all nuclei result smaller in the THT‐based electrolyte respect to both DMSO:THT and DMSO:TMS mixtures due to its higher viscosity (i. e., 10.10, 5.74, and 8.31 mPa· s for THT, DMSO:THT, and DMSO:TMS, respectively).
3. both Li and TFSI display very similar diffusivity in the two mixtures (DMSO:THT and DMSO:TMS) and almost double respect to THT solution, suggesting a strong effect of the ion′s solvation sphere.
4. the three systems exhibit the same relative order of diffusivity that is D_solv_>D_TFSI_>D_Li_, in agreement with previous findings concerning conventional organic electrolytes for LIBs.[^17^, ^50^, ^51^, ^52^, ^53^]

The Van der Walls radius of Li^+^ ion is between 0.073 and 0.090 nm and it is the smallest among the species in the solutions, but the hydrodynamic radius of the solvated Li^+^, from now on [Li(solv)_x_]^+^, is larger than those of the solvent molecules and the TFSI anion. It should be noted that NMR could not discriminate between free solvent molecules and bound ones in the [Li(solv)_x_]^+^ complexes, suggesting the lithium ions exchange ligands rapidly in the electrolytes.^[52]^

(8)

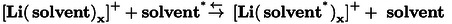




Consequently, the resulting D_solv_ is given as the weighted average value of both free and coordinated solvent to Li^+^ ions on the NMR time scale. However, the self‐diffusion coefficients of solvent molecules are always higher than lithium ones, suggesting that the fraction of solvents free from coordination prevails over that of solvating molecules.[^51^, ^54^, ^55^]

**Figure 7:**
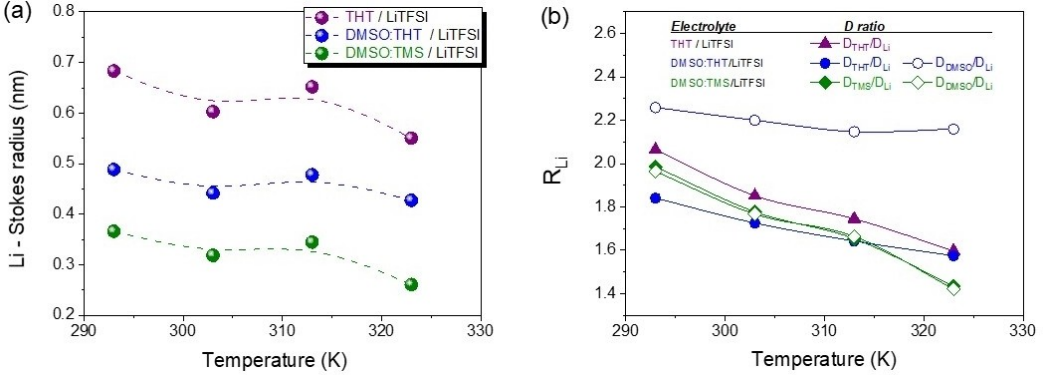
a) Stokes radii of lithium ion as a function of temperature obtained from the Stokes‐Einstein equation for the three solutions; b) Temperature variation in the range 293–363 K of R_Li_=r_solv_/r_Li_

The Stokes‐Einstein equation (Eq. 9) offers a foundational framework for understanding diffusion phenomena:

(9) $$D=\frac{k\;T}{6\pi \eta r_{s}}\;$$

where r_s_ is the effective hydrodynamic or Stokes radius, η is the viscosity, D is the self‐diffusion coefficient. This equation is used to estimate the lithium Stokes radius in all the electrolyte systems and the results are shown in Figure 7a. The values obtained ranged from 0.38 to 0.7 nm, indicating that the solvation of lithium is highly dependent on the solvent used. For instance, pure THT forms the largest coordination sphere (r_Li_ ∼ 0.7 nm at r.t.), followed by the mixtures DMSO:THT (r_Li_ ∼ 0.5 nm at r.t.), and finally by DMSO:TMS with the lowest Stokes radius (r_Li_ ∼ 0.38 nm at r.t.). This observation supports the evidence discussed in points 1 and 3 above. It is also noteworthy that in the DMSO:THT mixture the Stokes radius of lithium remains constant with temperature, unlike in the other two solutions, where it gradually decreases. This suggests partial desolvation of the cation due to thermal energy.

The Stokes equation can be further used to gain information about the contribution of each organic solvent molecule to the lithium coordination. Specifically, it can be readily derived that the ratio of the diffusion coefficients of the solvent to lithium is equal to the ratio of their Stokes radii (Equation 10).^[51]^ Figure 7b shows the temperature‐dependence of that ratios for the different solutions.

(10) $$\frac{D_{solv}}{D_{Li}}=\frac{r_{Li}}{r_{solv}}=R_{Li}$$

In THT electrolyte, R_Li_ is 2.1 at r.t, meaning that on average a lithium ion is coordinated by two solvent molecules in a sandwich‐like configuration.^[56]^ During the heating a partially desolvation occurs as the ratio decreases progressively.

The DMSO:THT mixture exhibits a significantly higher R_Li_ for DMSO (approximately 2.3) compared to THT (around 1.8 at r.t. ), suggesting that lithium coordination is primarily driven by DMSO, in line with the Raman study mentioned earlier. Furthermore, this coordination by DMSO appears stable and consistent across different temperatures, whereas the interaction with THT is weaker.

In contrast, in the DMSO:TMS mixture, both solvents contribute equally to cation coordination, with approximately 2 molecules of DMSO and 2 of TMS, and display even similar trend in temperature.

Similar approach used for the TFSI anion and discussed in the Supplementary Information (Figure S6), displays R_TFSI_ ranging between 1.2 and 1.4. Such values correspond to the ratio of van der Waal′s radii^[51]^ (between TFSI and each solvent molecule), indicating that the anion is not solvated but is stabilized through long‐range electrostatic interactions with the cation, as revealed by the Raman data.

The apparent lithium transport number (t_Li+_) can be calculated from the experimental self‐diffusion coefficients of Li^+^ and TFSI^−^ ions according to eq. (11) 11

(11) $$t_{{Li}^{+}}=\frac{D_{{Li}^{+}}}{D_{{Li}^{+}}+{\;D}_{{TFSI}^{-}}}$$

The three electrolyte solutions exhibit lithium transport numbers ranging from 0.36 to 0.39 at 20 °C, which are typical for organic aprotic electrolyte solutions..[^57^, ^58^, ^59^, ^60^, ^61^, ^62^]

**Figure 8:**
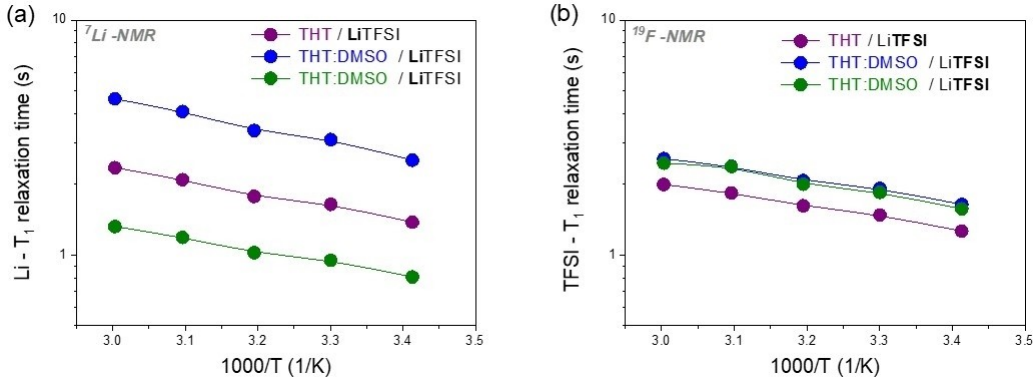
The temperature‐dependent behavior of T_1_ for (a) Li and (b) TFSI in the three electrolytes.

Finally, ^7^Li and ^19^F spin‐lattice relaxation times (T_1_) (Figure 8) can provide additional information on molecular‐scale interactions between ions and solvent molecules, since they are related to roto‐translational motions, reflecting how molecular movements affect the exchange of energy between nuclear spins and the lattice.

While the T_1_ values for TFSI are similar, as they correspond to the rotational motion of the CF_3_ groups, which are unaffected by interactions with various solvents or the lithium ion, the relaxation times of lithium, on the other hand, show a strong dependence on the solvent used.

The shortest T_1_ for lithium is observed in the DMSO:TMS mixture, suggesting that the short‐range motions of the cations are significantly restricted. This may be due to the formation of aggregates or clusters driven by strong electrostatic interactions with both the anion and solvent molecules. In contrast, the T_1_ values in the DMSO:THT mixture are significantly higher, indicating greater mobility of lithium and reduced ionic association. Such hypothesis will be confirmed in the following section on ionic association.

### 3.4 Ionic Conductivity and Association Degree

The ionic conductivity measured by impedance spectroscopy (equation 2, σ_EIS_) relies on the net migration of charged species (both cations and anions) within an electric field. Based on a Nernst‐Einstein assumption (Eq. 12), the ionic conductivity (σ_NMR_) can be calculated from the self‐diffusion coefficients of lithium ($D_{Li}$ ) and fluorine ($D_{TFSI}$ ):

(12) $$\sigma_{NMR}=\left(\frac{F^{2}c_{salt}}{R\cdot T}\right)\left(D_{Li}\;+D_{TFSI}\right)\;$$

where F is the Faraday constant and is the molar concentration of electrolytes. This equation considers all the species containing the NMR‐active nucleus, including isolated, ion‐paired, and solvated states. In other words, neutral aggregates do not contribute to charge transport because they do not migrate under the effect of the electric field, but they contribute to the diffusion. Therefore, σ_NMR_ is generally higher than σ_EIS_.

**Figure 9:**
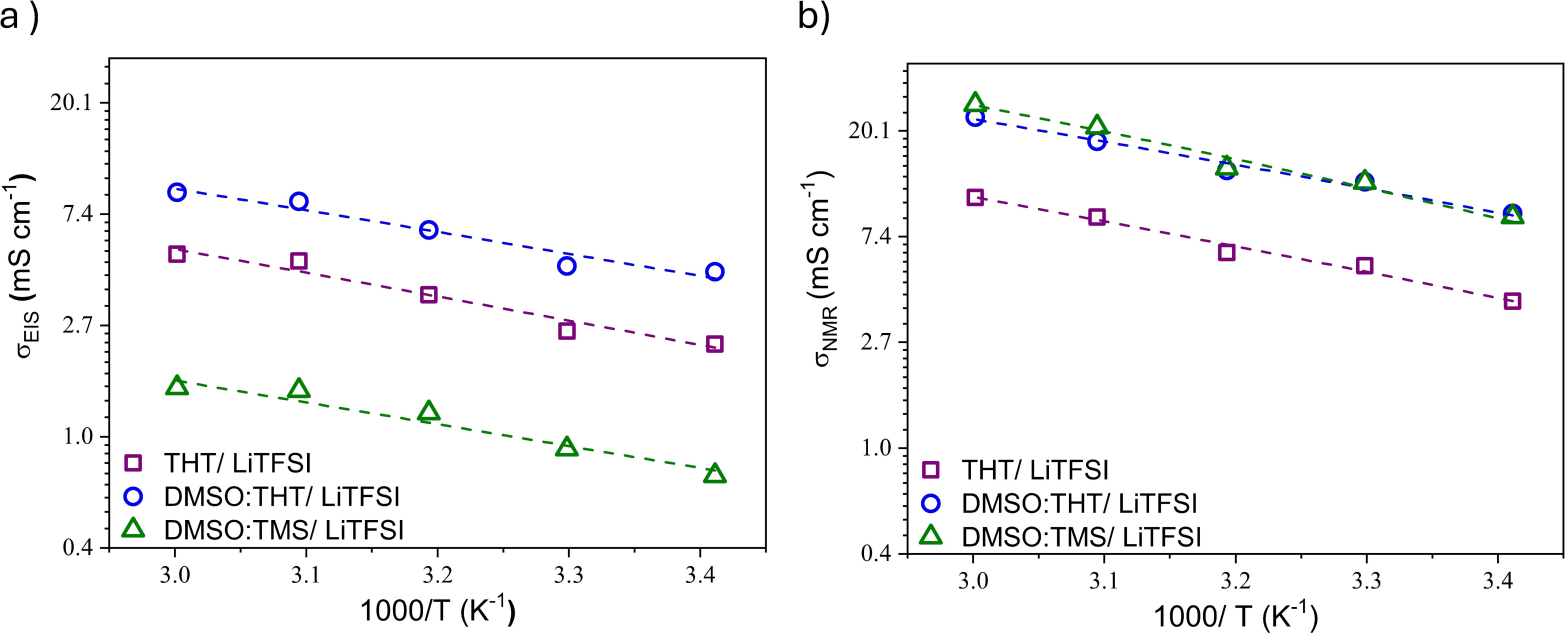
Ionic conductivity values (logarithmic scale) from a) EIS (σ_EIS_) and b) NMR (σ_NMR_) for the probed mixtures as a function of temperature. Dashed lines represent the fitting in terms of the VFT model.

Figure 9 shows both the Arrhenius plots of conductivities measured by EIS (σ_EIS_) and that estimated by the equation above (σ_NMR_). The temperature‐dependence of the conductivity was analyzed by VFT equation[^27^, ^28^, ^30^, ^33^, ^52^, ^57^, ^63^, ^64^] and the results are reported in the Supporting Information (Section S3, table S4), revealing a good linear decrease as function of T^−1^ for all electrolyte solutions.

The EIS data reveal the lowest conductivity for the DMSO:TMS system, while the highest conductivity is recorded in the system where DMSO is mixed with THT.

In complex electrolytic systems like those under study, strong dipolar interactions between solvent molecules weaken the solvation forces acting on the cations, leading to ionic association. This can result in the formation of aggregates, which may be neutral (anion‐cation pairs) or take the form of larger ionic clusters (such as triplet of ions). Even if these aggregates are charged, their mobility is reduced compared to individual ions due to larger hydrodynamic radius. Additionally, the interactions between cations and anions may shield part of the internal charge, further decreasing their contribution to charge transport. In both cases, ionic association leads to lower ionic conductivity, thus contributing to the observed gap between the results of the two techniques (EIS, NMR).

**Figure 10:**
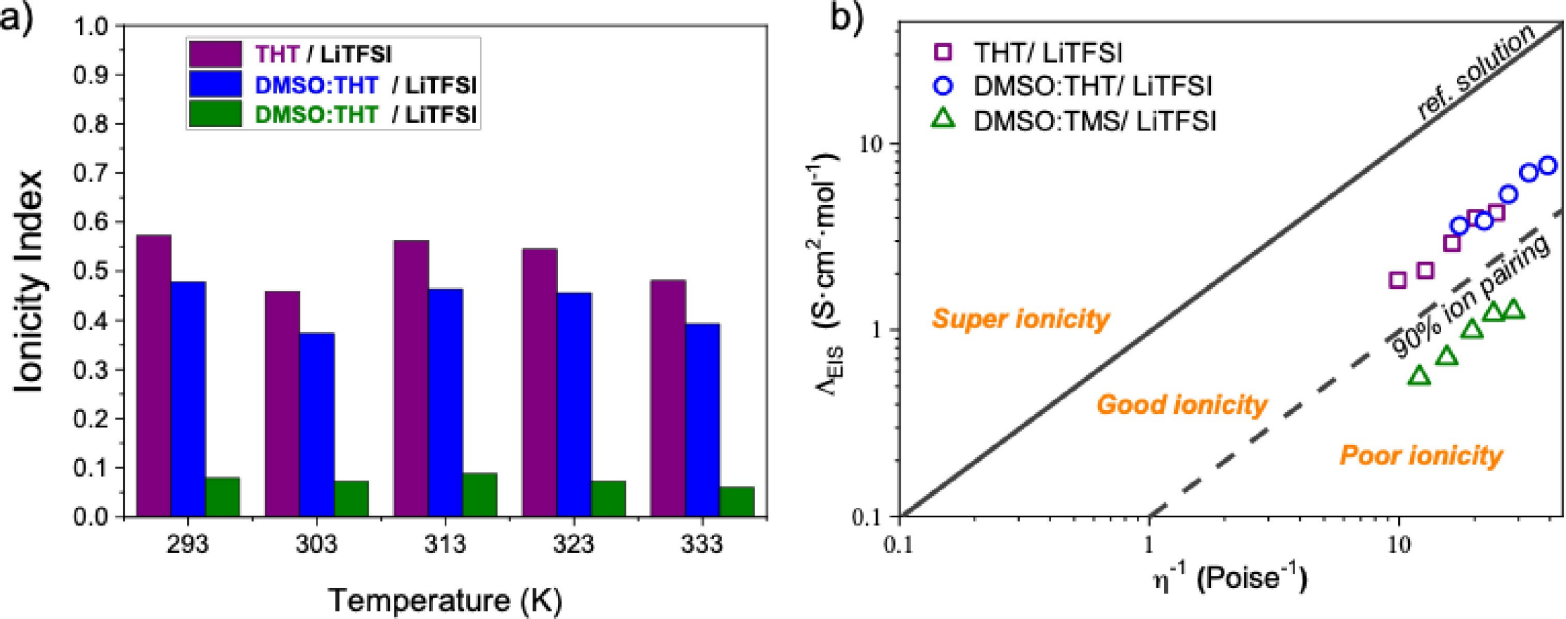
a) Ionicity index for THT (purple), DMSO:THT (blue), and DMSO:TMS (green) electrolytes in the temperature range 293–363 K. b) Walden plot (log (Λ) vs. log (η^−1^)) for the probed electrolytes at different temperatures considering EIS results. The central line represents the ideal trend for a 0.01 mol L^−1^ aqueous KCl solution while the dashed one indicates an ion pairing of 90 %.

It is commonly accepted that the degree of dissociation of an electrolyte can be estimated from the ratio of σ_EIS_ to σ_NMR_ (σ_EIS_/σ_NMR_). The degree of dissociation, also known as the ionicity index, for all three electrolyte systems is shown in Figure 10a as a function of temperature. It can be seen that the ionicity index for THT and DMSO:THT electrolytes ranges from 0.57 to 0.45, which are typical values for conventional organic electrolytes.[^51^, ^65^] Conversely, the DMSO:TMS electrolyte displays a notably low ionicity (approximately 0.1), indicating a high concentration of ionic aggregates, which explains its reduced conductivity. From these data, we can conclude that TMS promotes ionic association, likely due to its ability to enhance solvent‐solvent dipolar interactions.

Finally, conductivity values can be correlated with dynamic viscosity. Molar conductivity (Λ=σ/M_salt_, where M_salt_ represents the salt concentration expressed in mol L^−1^) is presented in Figure 10b as a function of fluidity (η^−1^),^[27]^ according to the Walden product as described in the equation 13 by the Walden rule: 13

(13) Λ·η=k

where k is a temperature‐dependent constant.

The Walden plot[^66^, ^67^, ^68^] analysis provides insights into the degree of ionic dissociation within a mixture, compared to an idealized reference trend established by dilute aqueous KCl solutions, which assume the presence of independent ions without interionic interactions. The Walden plot typically features three regions, delineated by two reference lines. The solid line represents the behaviour of the reference solution (0.01 M KCl_aq_). The dashed line indicates 90 % ion pairing. Systems located between these lines exhibit *good ionicity*, while those above the solid line or below the dashed one are considered with a *super ionicity* and *poor ionicity*, respectively. This plot corroborates the above discussion, confirming once again that the DMSO:TMS mixture exhibits poor ionicity.

## 4 Conclusions

In this study, we conducted a detailed analysis of the physical, structural, dynamic, and transport properties of 1m solutions of LiTFSI in three different sulfur‐based solvent systems: THT/LiTFSI, DMSO:THT/LiTFSI and DMSO:TMS/LiTFSI. A thorough understanding of how different solvents influence lithium transport within the bulk is crucial for optimizing battery performance and guiding the targeted design of electrolyte components. The combined analysis of spectroscopic and electrochemical data provides complementary information on the internal dynamics of these systems.

1. NMR and Raman analyses showed that, in the absence of an electric field, solvent molecules establish weak interactions with lithium ions, allowing TFSI^−^ to enter the cation coordination shell due to the predominant Coulombic interactions. The shift of the peaks related to the S−N−S vibration of the TFSI^−^ suggests the formation of contact ion pairs and larger ionic complexes in the solutions studied and was confirmed by suitably computed Raman spectra.
2. The application of an electric field to the electrolytes to enable directional ion motions, as investigate by EIS measurements, showed significant variations in ionic conductivity between the different electrolytes.
3. In the DMSO:TMS/LiTFSI electrolyte, solvent molecules are less effective in the lithium solvation process, leading to the formation of complex and less dynamic solvation structures. The presence of strong interactions between Li and the TFSI, together with the relatively high viscosity of the system, further limits the lithium mobility. The combination of these factors hinders the efficiency of ion transport, contributing to the lower performance of the system compared to other electrolyte solutions studied.
4. The THT/LiTFSI electrolyte shows good ionic conductivity, despite the intrinsic viscosity of solvent. This result is attributable to the rapid exchange mechanism between solvent molecules interacting with lithium in a sandwich‐type structure, favoring a more dynamic ionic transport as well as the highest ionicity. This structure creates a less coordinating environment that is also accessible to TFSI^−^, which improves ionic mobility. The stability of this configuration was evident both under temperature conditions (Figure S2) and under the action of an electric field (Figure 9).
5. In the DMSO:THT/LiTFSI electrolyte, the combination of the two solvents proved to be particularly advantageous. DMSO reduces the overall viscosity of the system, decreasing the resistance to ion movement and improving the friction in the bulk. At the same time, THT maintains a dynamic mechanism of rapid exchange between solvent molecules, improving the dynamic and ion transport properties, as confirmed by the higher ionic conductivity and diffusivity observed. Lithium perceives a less coordinating environment, as evidenced by the T_1_ relaxation times and Raman data, which show a reduction in ion pairs and aggregates. This combination of DMSO and THT creates an ideal balance between fluidity and dynamism.

As a final remark, we would like to underline that this study about transport mechanism of ions in sulphones/sulphoxides facilitates the way for a rational implementation of S‐based solvents in aprotic electrolytes, to go beyond the use of the state‐of‐the‐art organic carbonates in aprotic lithium batteries. Overall, sulphones can implement the functional properties of aprotic electrolytes by: (a) broadening the operating temperature range;^[69]^ (b) mitigating the side reactions upon cycling thus improving the overall battery safety^[70]^ as well as (c) widening the electrochemical stability window allowing for a larger operating voltage range and therefore improving the energy density of the battery device.^[71]^ To this aim, our study demonstrated the complexity of the interplay between solvent molecules like DMSO and THT with the LiTFSI salt and proved their unexpected synergistic behavior in the mixed electrolyte (i. e. DMSO:THT/LiTFSI) where the mobility of ions is enhanced by the reduced viscosity and the fast Li^+^ exchange mechanism among solvent species.

## Ackowledgements

This work has been financed by the Research Fund for the Italian Electrical System under the Three‐Year Research Plan 2022–2024 (DM MITE n. 337, 15. 09. 2022), in compliance with the Decree of April 16th, 2018. The research project here reported was also supported by the “Centro Nazionale per la Mobilità Sostenibile (MOST) CN4 Spoke 13 Batterie e Trazione Elettrica” funded by the Italian Government and the European Union in the frame of the “Missione 4 Componente 2 Investimento 1.4 – Potenziamento strutture di ricerca e creazione di “campioni nazionali di R&S” su alcune Key Enabling Technologies del PNRR (Avviso MUR n.3138 del 16–12‐2021)” prot. Sapienza CN4621845C7D1585 and SUDELBAT project (CN00000023, CUP D43 C22001180001).

## Conflict of Interests

The authors declare no conflict of interest.

## Supporting information

As a service to our authors and readers, this journal provides supporting information supplied by the authors. Such materials are peer reviewed and may be re‐organized for online delivery, but are not copy‐edited or typeset. Technical support issues arising from supporting information (other than missing files) should be addressed to the authors.

Supporting Information
