# Supplementary material for: Aprotic Electrolytes Beyond Organic Carbonates: Transport Properties of LiTFSI Solutions in S‐Based Solvents
Source: ChemSusChem. 2025 Jan 17;18(9):e202402273. doi: 10.1002/cssc.202402273 (PMC12051247; doi:10.1002/cssc.202402273)
Supplement: Supplementary file 1 — Supporting Information [file CSSC-18-e202402273-s001.pdf]

ChemSusChem

Supporting Information

Aprotic Electrolytes Beyond Organic Carbonates: Transport Properties of LiTFSI Solutions in S-Based Solvents

Vanessa Piacentini, Cataldo Simari,* Emanuela Mangiacapre, Adriano Pierini, Antonio Gentile, Stefano Marchionna, Isabella Nicotera, Sergio Brutti, and Enrico Bodo*

Supporting information

Aprotic electrolytes beyond organic carbonates: transport properties of LiTFSI solutions in S-based solvents

S1. Physical characterization

Table S1. Fitting parameters of the experimental density for the probed electrolytes as a function of temperature, in terms of the model $\rho(T)=AT^2+BT+\rho_o$.

Electrolyte (1m LiTFSI)	THT	DMSO:THT	DMSO:TMS
**A [g·cm^{-3}·K^{-2}]**	2.03E-08	-8.96E-08	-2.39E-07
**B [10^{-4} ·g·cm^{-3}·K^{-1}]**	-9.1E-4	-9E-4	-7.9E-4
**ρ_o [g/cm^3]**	1.51	1.49	1.52

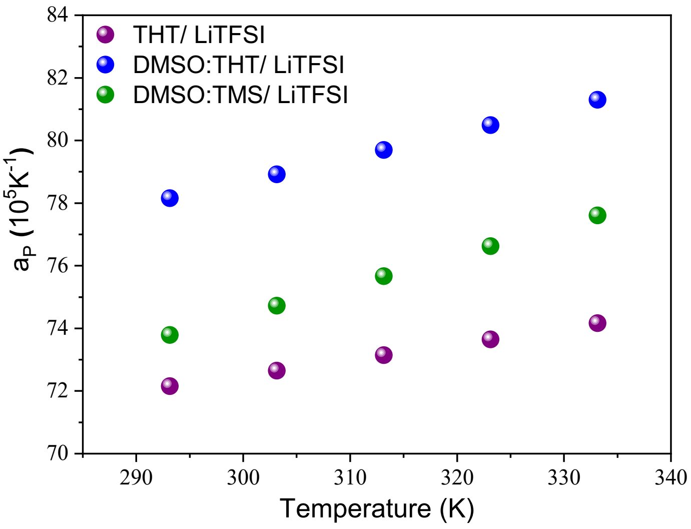

Figure S1. Isobaric thermal expansivity as function of temperature between 293 and 333K for the probed electrolytes.

Table S2. Fitting parameters of the isobaric thermal expansion coefficients for the probed electrolytes as function of temperature using a linear model: $\alpha_P = A + B \cdot T$.

Electrolyte (1m LiTFSI)	THT	DMSO:THT	DMSO:TMS
**A [10^5 K^{-1}]**	57.43	55.14	45.80
**B [K^{-1}]**	0.0502	0.0785	0.0954

Table S3. Fitting parameters of the experimental dynamic viscosity for the probed electrolytes as a function of temperature, in terms of the Vogel-Fulcher-Tammann model.

Electrolyte (1m LiTFSI)	THT	DMSO:THT	DMSO:TMS
**ln(η_0)**	-3.600	-2.849	-2.149
**B [K]**	1299	853.3	669.4
**T_0 [K]**	73.50	107.6	136.3

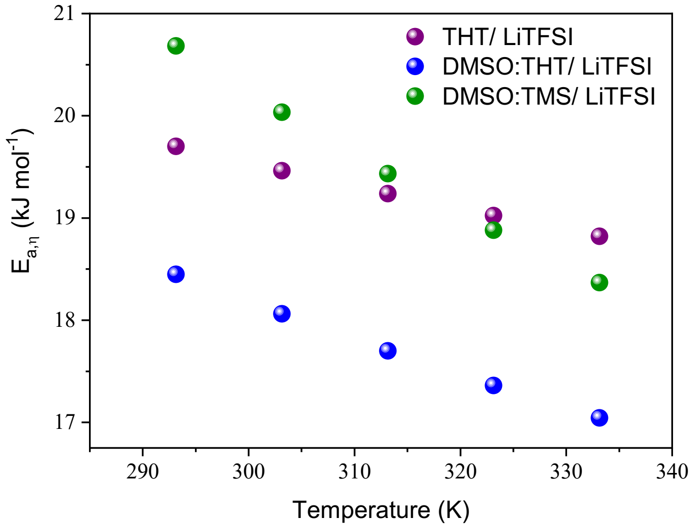

Figure S2. Activation energy for viscous flow for the probed electrolytes as function of temperature.

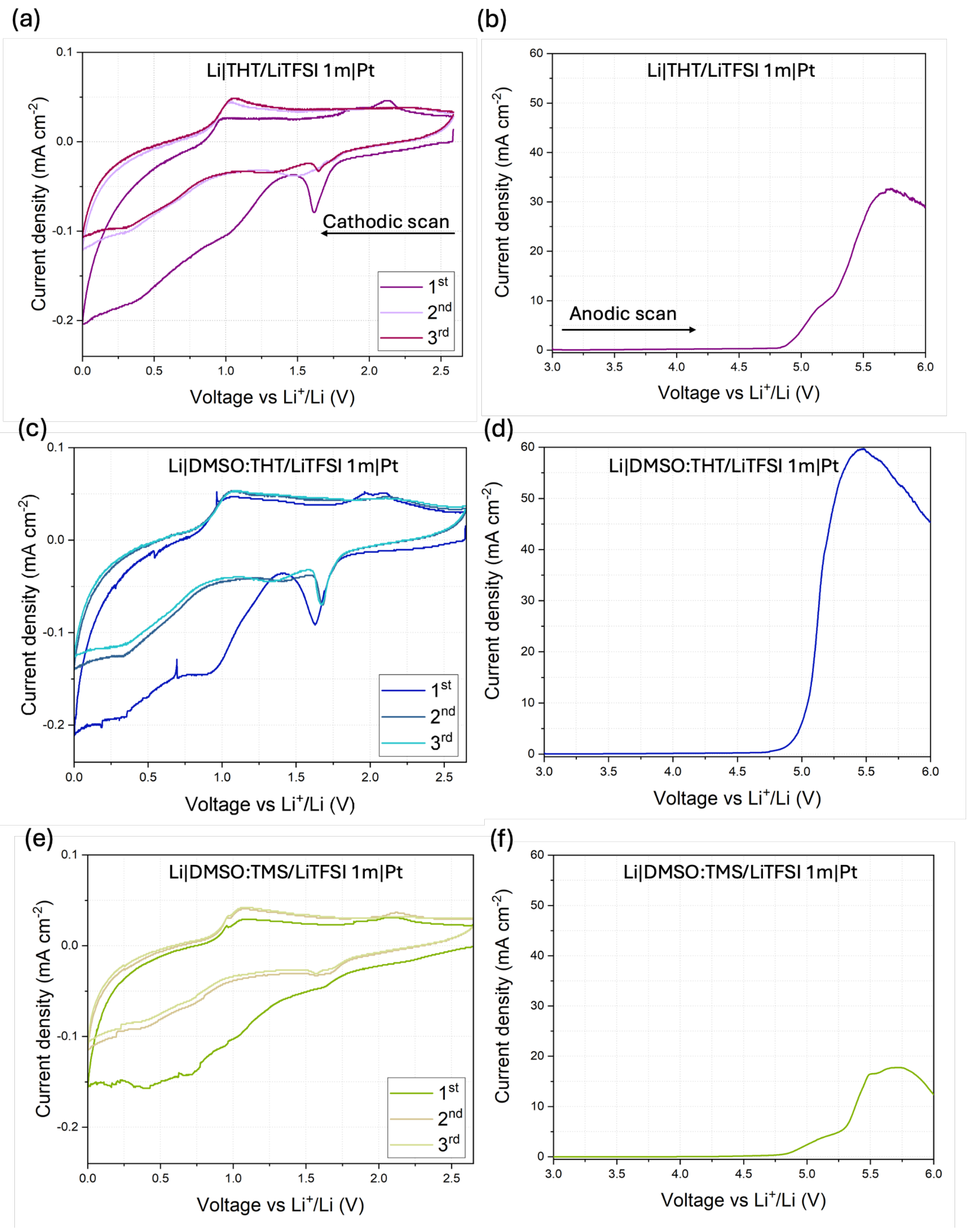

Figure S3: Cyclic Voltammetry and Linear Sweep Voltammetry of THT/ LiTFSI 1m (a,b), DMSO:THT/ LiTFSI 1m (c,d) and DMSO:TMS/ LiTFSI 1m (e,f).

S3. Raman analysis

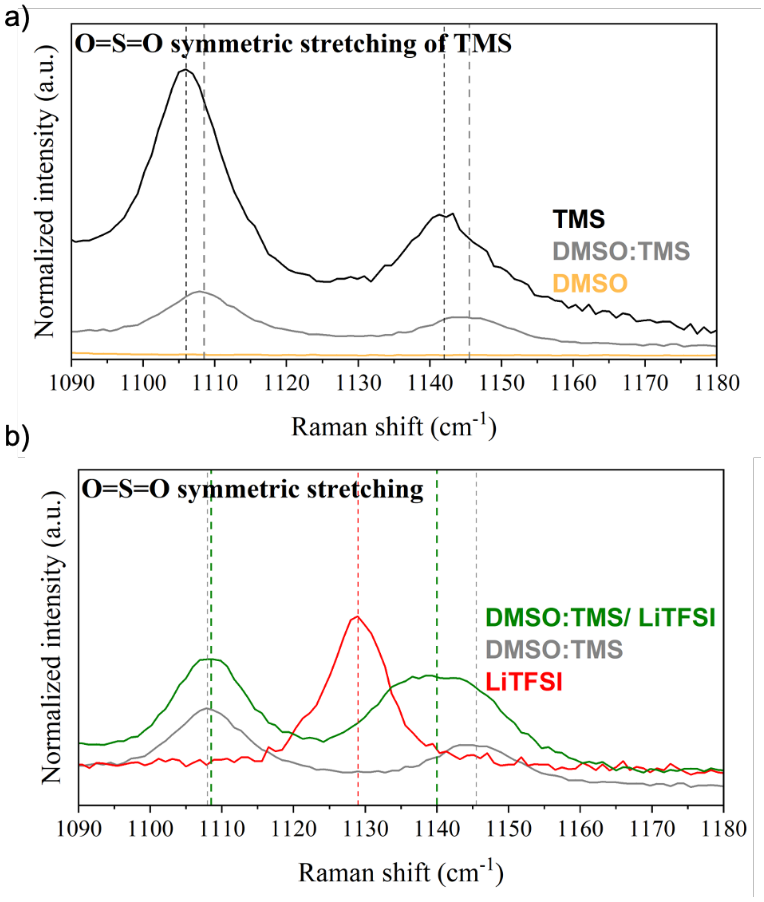

Figure S4: a) Raman spectra in the SO_2 stretching region of neat DMSO, neat TMS and DMSO:TMS mixture; b) Raman spectra of the SO_2 stretching region of pure TFSI, in DMSO:TMS, electrolyte solution.

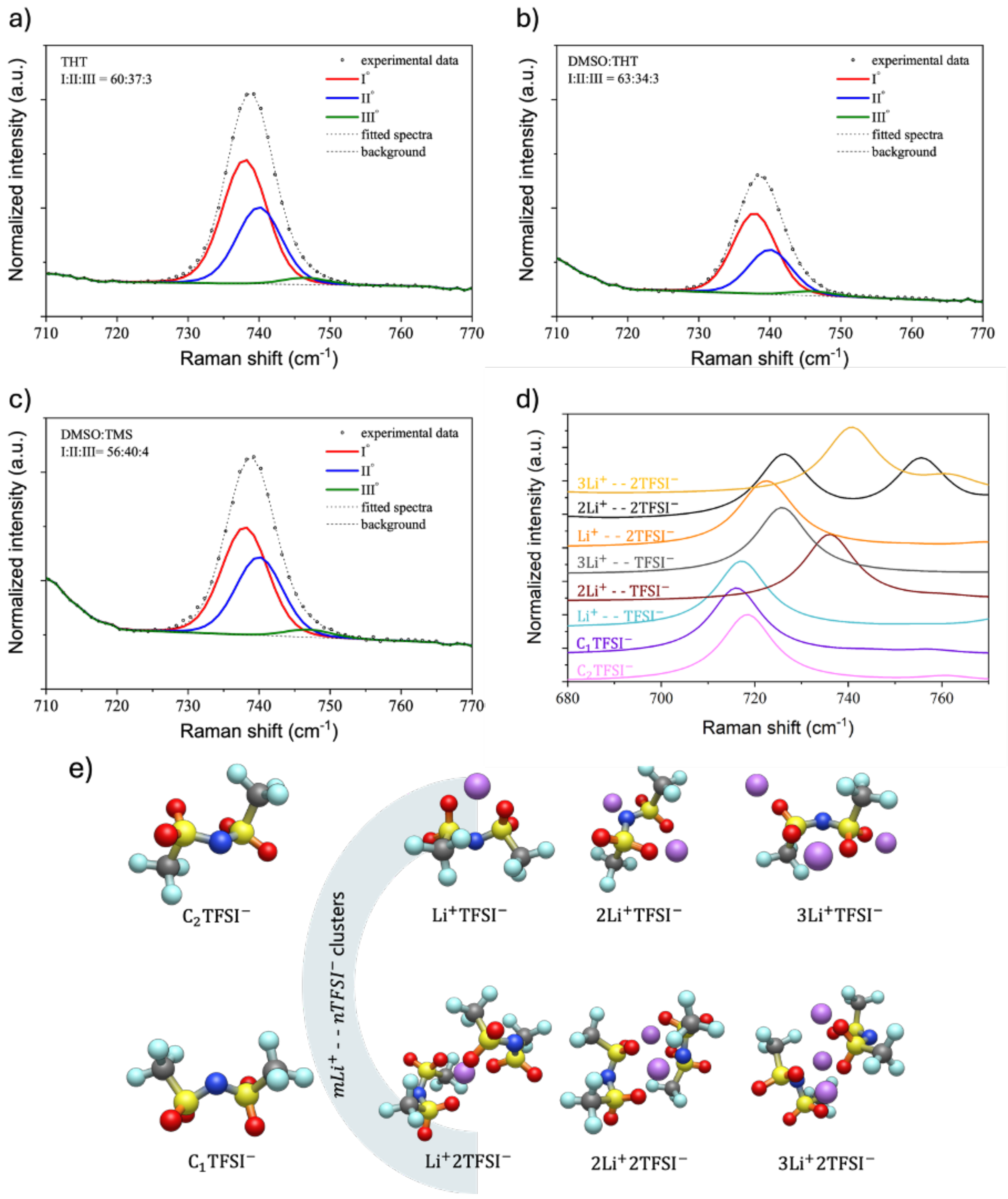

Figure S5: Deconvolution of the Raman band associated with the S-N-S vibration of the TFSI anion for a) THT b) DMSO:THT c) DMSO:TMS; d) theoretical Raman spectrum for the S-N-S vibrational bands of the clusters $m\mathrm{Li}^{+}n\mathrm{TFSI}^{-}$ and e) their DFT-optimized geometries.

Discussion of Figure S5

The Raman spectra shown in Figure S5 have been computed for different neutral and ionic structures that have been chosen because they appear to be representative of the salt aggregation state according to ref. 12 of the main text. These encompass the isolated anion (for reference), the neutral ionic couple $\mathrm{Li^{+}TFSI^{-}}$, three cationic species $\mathrm{2Li^{+}TFSI^{-}}$, $\mathrm{3Li^{+}TFSI^{-}}$, $\mathrm{3Li^{+}2TFSI^{-}}$, a neutral cluster $\mathrm{2Li^{+}2TFSI^{-}}$ and the anionic variant $\mathrm{Li^{+}2TFSI^{-}}$. Computational absorption frequencies are obtained in the harmonic approximation using the Hessian computed in the local minima. Hence, they need to be scaled to account for anharmonicity effects. The scaling factor chosen by us is 0.97 that is quite typical for the specific combination of functional and basis set (see ref 24 of main text).

The curves in panel (d) of Figure S4 are the theoretical spectral profiles as obtained by adding a Gaussian broadening to the computed frequency of the vibrational normal mode dominated by the motion of the S-N-S group. They show that the frequencies of the S-N-S vibrational mode is below 730 cm^{-1} for a “free” TFSI anion (pink and purple lines). This should exclude the presence of "free" TFSI anions in the electrolyte solutions. Taking solvation into account, the frequency would undergo an additional red shift. The vibrational frequencies of the neutral ionic couple $\mathrm{Li^{+}TFSI^{-}}$ (cyan) and of the anionic cluster $\mathrm{Li^{+}2TFSI^{-}}$ (orange) also are red shifted with respect to the experimental signal as it is also the frequency of the doubly charged $\mathrm{3Li^{+}TFSI^{-}}$ complex (grey). The possible candidates for the experimental band at 740-750 cm^{-1} that are left are the singly charged cluster $\mathrm{3Li^{+}2TFSI^{-}}$ (yellow) and $\mathrm{2Li^{+}TFSI^{-}}$ (brown) and the neutral $\mathrm{2Li^{+}2TFSI^{-}}$ specie (black). The latter however shows the appearance of a two separate signals at 720-730 and 750-760 cm^{-1} that lie in region where the experimental spectra is essentially zero. The near-perfect alignment between the computed frequencies of two cationic clusters $\mathrm{3Li^{+}2TFSI^{-}}$ (yellow) and $\mathrm{2Li^{+}TFSI^{-}}$ (brown) and the dominant red and blue deconvoluted experimental bands in panels (a–c) suggests that they might represent, possibly, the primary forms of aggregation in the electrolytes. These findings are consistent with observations reported in Ref. 41 (see main text), that discussed the presence of cationic Li-TFSI aggregates and their characteristic Raman spectral signatures. However, we stress that the variety, and structural mutability of coordination patterns exhibited by these species makes a definitive structural characterization particularly challenging.

S3. *Multinuclear NMR characterization and EIS*

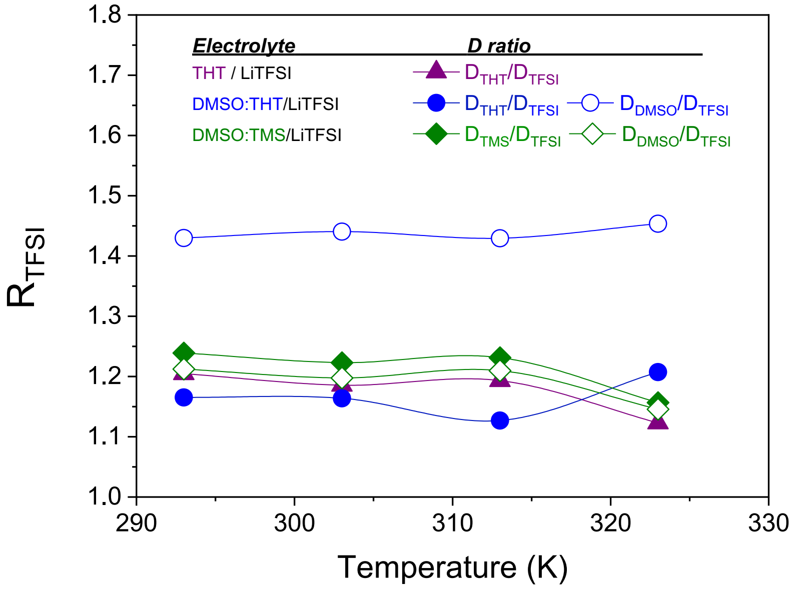

Figure S6. The R values of the $TFSI^-$ for the electrolytes vs the temperature. See eq (1) in main text

The temperature-dependence of the conductivity was fitted by VFT equation:

$$ln(\sigma) = ln[\![(\sigma]\!]_0) + \frac{D_\sigma}{T - T_0}$$

where σ_0 is the constant of pre-exponential factor, D_σ is the conductometric apparent activation energy and T_0 is the temperature at which the conductivity equals to zero (fitting parameters are reported in table S4). According to the VFT equation, the $\ln(\sigma)$ should be linearly related to T^{-1}.

Table S4. Fitting parameters of the experimental EIS and NMR ionic conductivity for the probed electrolytes as a function of temperature, in terms of the Vogel-Fulcher-Tammann model.

**EIS**			
System	THT	DMSO:THT	DMSO:TMS
ln (σ_0)	7.051	7.205	6.262
D	-1521	-1431	-1879
T_0 (K)	49.85	45.72	6.926

**NMR**			
System	THT	DMSO:THT	DMSO:TMS
ln (σ_0)	7.370	7.611	7.651
D	-1220.936	-1072.394	-882.464
T_0 (K)	88.913	94.971	132.961
